# 1918 pandemic morbidity: The first wave hits the poor, the second wave hits the rich

**DOI:** 10.1111/irv.12541

**Published:** 2018-02-06

**Authors:** Svenn‐Erik Mamelund

**Affiliations:** ^1^ Work Research Institute OsloMet‐Oslo Metropolitan University Oslo Norway

**Keywords:** 1918‐19 influenza pandemic, apartment size, cross‐protection, gender, influenza‐like illness, morbidity crossover, socioeconomic status

## Abstract

**Background:**

Whether morbidity from the 1918‐19 influenza pandemic discriminated by socioeconomic status has remained a subject of debate for 100 years. In lack of data to study this issue, the recent literature has hypothesized that morbidity was “socially neutral.”

**Objectives:**

To study the associations between influenza‐like illness (ILI) and socioeconomic status (SES), gender, and wave during the 1918‐19 influenza pandemic.

**Methods:**

Availability of incidence data on the 1918‐19 pandemic is scarce, in particular for waves other than the “fall wave” October‐December 1918. Here, an overlooked survey from Bergen, Norway (n = 10 633), is used to study differences in probabilities of ILI and ILI probability ratios by apartment size as a measure of SES and gender for 3 waves including the waves prior to and after the “fall wave.”

**Results:**

Socioeconomic status was negatively associated with ILI in the first wave, but positively associated in the second wave. At all SES levels, men had the highest ILI in the summer, while women had the highest ILI in the fall. There were no SES or gender differences in ILI in the winter of 1919.

**Conclusions:**

For the first time, it is documented a crossover in the role of socioeconomic status in 1918 pandemic morbidity. The poor came down with influenza first, while the rich with less exposure in the first wave had the highest morbidity in the second wave. The study suggests that the socioeconomically disadvantaged should be prioritized if vaccines are of limited availability in a future pandemic.

## 1 BACKGROUND

The 1918‐19 pandemic killed 50‐100 million during 3 waves.^1^ The 1970‐2000 literature claimed that this pandemic infected and killed all classes equally.^2^, ^3^, ^4^, ^5^ However, later studies questioned the “socially neutral view” regarding *mortality* and found higher mortality for the poor along several socioeconomic indices including countries and income per head,^1^, ^6^ boroughs,^7^, ^8^, ^9^, ^10^ occupational classes and apartment size,^8^ literacy, homeownership, and unemployment.^11^

Contemporary surveys found mixed associations between socioeconomic status (SES) and *morbidity*. First, controlling for age, gender, and race using data from 9 US cities in the fall of 1918, a negative association between an individual's economic status (very poor, poor, moderate, and well‐to‐do) and morbidity and a positive association between persons per room and morbidity were found.^12^ Second, a bivariate study of Bergen, Norway, analyzing 3 waves in combination, found a moderate negative association between number of rooms and morbidity.^13^ Third, bivariate studies of 5 English cities, combining data for 3 waves, found no association between persons per room and morbidity in 3 of the cities and a positive correlation in 2.^14^ Finally, a bivariate study of Boston with data from fall of 1918 found no difference in morbidity by SES of districts (very poor, poor, moderate, well‐to‐do), but persons per room and cleanliness (very dirty, dirty, clean, very clean) were respectively positively and negatively associated with morbidity.^15^ Without having data to analyze the link between SES and morbidity, recent studies cite 2 of these surveys^14^, ^15^ and conclude that morbidity was not associated with SES.^8^, ^16^, ^17^, ^18^

Here, the association between apartment size and 1918‐19 pandemic influenza‐like illness (ILI) is analyzed using overlooked aggregate survey data from Bergen, Norway.^13^ The aims of this article were (i) to analyze the associations between apartment size and ILI by gender and 3 waves; (ii) to analyze gender differences in ILI by apartment size within each wave; and (iii) to assess the association between first‐wave ILI by gender and apartment size and ILI during the subsequent waves. Surveys for other locations cannot be used for this analysis because data at the time were collected only for the wave in the fall of 1918,^12^, ^15^ or when data were collected for 3 waves, data were published for the entire pandemic period.^14^

Research on the SES drivers of morbidity and cross‐protection across waves has recently been called for,^19^, ^20^ but not carried out due to a lack of data, and is important for several reasons. First, knowing whether specific SES groups have higher morbidity can help targeting scarce pandemic vaccines, and thus reduce the human, social, and financial losses in the next pandemic. Second, studies of morbidity can help understand whether SES differences in mortality reported in prior studies are due to SES differences in exposure/morbidity, case fatality, or both. Finally, the topic is timely with the centenary of the 1918 pandemic in 2018.

In this study, number of rooms is a measure of SES and a mechanism for exposure to aerosol/airborne diseases such as influenza. It is hypothesized that low SES > larger families > smaller apartments > more persons per room > higher exposure > higher influenza morbidity.

## 2 MATERIALS AND METHODS

The data derive from a survey of the pandemic in Bergen, Norway.^13^ Eight boroughs were selected randomly. This strategy secured both households with and without influenza cases. The at‐risk age distributions and the influenza mortality in the sample and the population were the same.^13^, ^21^

All households were interviewed by trained nurses from the end of 1918 to the end of 1919, and the data were categorized and published along the observed waves July‐September 1918, October‐December 1918, and January‐March 1919. The ILI cases and deaths were self‐reported by a homemaker.

The sample consists of 10 633 individuals, 4818 cases, and 72 deaths and covers 11.8% of the population. The individual‐level data are lost, but aggregate outcome data by apartment size, gender, and wave were published and used here.^13^ Although data on outcomes and population at risk are also available by, respectively, age, gender, and wave, and by the 8 sample boroughs, gender, and wave, the data on age and boroughs cannot be combined with the data on apartment size, gender, and wave. Death and fatality rates are not analyzed. Results not shown produced no significant differences in mortality by apartment size, gender, and wave due to few sample deaths. However, “signature features,” *substantially* higher mortality for men, W‐shaped mortality, and highest mortality in the second wave were documented just as in prior studies.^21^

The outcome variable is the probability of reporting an influenza‐like illness (ILI) during each wave, that is, ILI cases in % of the risk population at the start of the wave considered. The population at risk at the start of the fall and winter waves are respectively adjusted for cases during the summer and fall waves. As 6.5% of the respondents reported re‐infection during the fall and winter waves,^13^ only a factor of 0.935 of the summer‐wave and fall‐wave cases is subtracted from the population at risk at the start of the fall wave and winter wave, respectively.

The explanatory variables are SES, gender, and wave. The SES measure is number of rooms in an apartment. The apartment categories are as follows (% of sample): 1 room with/without kitchen (31%), 2 rooms with kitchen (31%), 3 rooms with kitchen (15%), and 4 or more rooms with kitchen (22%). Because only 10 men and 13 women lived in apartments with 1 room without kitchen (and with only 1 female and 1 male ILI case in the summer wave, and with no other cases throughout the pandemic), they were analyzed together with those living in 1‐room apartments with kitchen. The analysis was carried out for the 3 waves described above.

Differences in ILI were estimated using probability differences and ratios. A statistically significant difference was estimated using a 1‐sided *z* test with α = 5%, that is, *z* ≥ 1.65 (when alpha is 10%, 1%, or 0.1%, then the *z*‐values are ≥1.29, ≥2.33, and ≥3.12, respectively). The associations between first‐wave ILI and illness in subsequent waves were estimated using Pearson correlations and a *t* test.

## 3 RESULTS

Half of the sample (45.3%) reported having had an ILI 1918‐19 (Table 1). Results strongly suggest that women living in 2‐room apartments had higher morbidity (significant at 10%). If we compare the 2 smallest apartment types with the 2 largest and disregard gender, morbidity was significantly lower among residents of larger apartments (significant at 5%, *z* = −1.92).

**Table 1:** Morbidity, as percentage suffering from an influenza‐like illness, by size of apartment and gender during all 3 waves, July 1918‐March 1919

Size of apartment	Risk population	ILI cases	Probability of ILI (ILI cases in % of risk population)	Probability differences (95% CI)	*z*	Probability ratios
Both genders						
1 room	3 356	1 524	45.41	Reference		(ref.) 1.00
2 rooms	3 287	1 582	48.13	2.72 (−2.09 to 7.53) n.s.	1.11	1.06
3 rooms	1 664	713	42.85	−2.56 (−8.44 to 3.31) n.s.	−0.85	0.94
4 rooms +	2 326	999	42.95	−2.46 (−7.75 to 2.83) n.s.	−0.91	0.95
Men						
1 room	1 526	719	47.12	Reference		(ref.) 1.00
2 rooms	1 584	747	47.16	0.04 (−6.99 to 7.07) n.s.	0.01	1.00
3 rooms	726	318	43.80	−3.31 (−12.15 to 5.52) n.s.	−0.74	0.93
4 rooms +	898	384	42.76	−4.35 (−12.60 to 3.89) n.s.	−1.04	0.91
Women						
1 room	1 830	805	43.99	Reference		(ref.) 1.00
2 rooms	1 703	835	49.03	5.04 (−1.56 to 11.64)*	1.50	1.11
3 rooms	938	395	42.11	−1.88 (−9.75 to 5.99) n.s.	−0.47	0.96
4 rooms +	1 428	615	43.07	−0.92 (−7.84 to 6.00) n.s.	−0.26	0.98

*α = 10%, *z* ≥ 1.29.

The entire pandemic period masks fundamental differences. During the summer, both genders combined and men residing in, respectively, 3‐ and 4‐room apartments had significantly *lower* ILI (Table 2). Although not significant, results for women also go in the same direction (significant at 10%). One in 3 and 1 in 5 in the 2 smallest and 2 largest apartment categories, respectively, had ILI: These differences were significant at the 0.1% level for men (*z* = −3.17) and women (*z* = −2.93), and at the 1% level for both genders (*z* = −4.38). Results also suggest that women living in 2‐room apartments had higher ILI (significant at 10%).

**Table 2:** Morbidity, as percentage suffering from an influenza‐like illness, by size of apartment and gender during the first wave, July‐September 1918

Size of apartment	Risk population	ILI cases	Probability of ILI (ILI cases in % of risk population)	Probability differences (95% CI)	*z*	Probability ratios
Both genders						
1 room	3 356	902	26.88	Reference		(ref.) 1.00
2 rooms	3 287	1 028	31.27	4.40 (−0.41 to 9.21)*	1.79	1.16
3 rooms	1 664	339	20.37	−6.50 (−12.38 to 0.63)*	−2.17	0.76
4 rooms +	2 326	470	20.21	−6.67 (−11.96 to 1.38)**	−2.47	0.75
Men						
1 room	1 526	445	29.16	Reference		(ref.) 1.00
2 rooms	1 584	514	32.45	3.29 (−3.74 to 10.32) n.s.	0.92	1.11
3 rooms	726	164	22.59	−6.57 (−15.41 to 2.27)***	−1.46	0.77
4 rooms +	898	179	19.93	−9.23 (−14.47 to 0.98)*	−2.19	0.68
Women						
1 room	1 830	457	24.97	Reference		(ref.) 1.00
2 rooms	1 703	514	30.18	5.21 (−1.39 to 11.81)***	1.55	1.21
3 rooms	938	175	18.66	−6.32 (−14.19 to 1.55)***	−1.57	0.75
4 rooms +	1 428	291	20.38	−4.59 (−11.52 to 2.33)***	−1.30	0.82

*α = 5%, *z* ≥ 1.65; **α = 1%, *z* ≥ 2.33; ***α = 10%, *z* ≥ 1.29.

A crossover in morbidity by apartment size occurred moving from the summer to the fall. In the fall, gradually more men and women reported an ILI by apartment size (Table 3). The trend is clear, and there is a tendency that those living in apartments with >4 rooms disregarding gender had the highest morbidity (significant at 10%).

**Table 3:** Morbidity, as percentage suffering from an influenza‐like illness, by size of apartment and gender during the second wave, October‐December 1918

Size of apartment	Risk population	ILI cases	Probability of ILI (ILI cases in % of risk population)	Probability differences (95% CI)	*z*	Probability ratios
Both genders						
1 room	2 513	381	15.16	Reference		(ref.) 1.00
2 rooms	2 326	417	17.93	2.77 (−2.87 to 8.41)	0.96	1.18
3 rooms	1 347	240	17.82	2.65 (−3.97 to 9.27)	0.79	1.17
4 rooms +	1 887	370	19.61	4.55 (−1.52 to 10.42)*	1.46	1.29
Men						
1 room	1 110	165	14.87	Reference		(ref.) 1.00
2 rooms	1 103	175	15.86	0.99 (−7.34 to 9.33) n.s.	0.23	1.07
3 rooms	573	104	18.16	3.29 (−6.79 to 13.38) n.s.	0.64	1.22
4 rooms +	731	141	19.30	4.43 (−4.91 to 13.77) n.s.	0.93	1.30
Women						
1 room	1 403	216	15.40	Reference		(ref.) 1.00
2 rooms	1 222	242	19.80	4.40 (−3.27 to 12.07) n.s.	1.12	1.29
3 rooms	774	136	17.56	2.16 (−6.61 to 10.94) n.s.	0.48	1.14
4 rooms +	1 156	229	19.81	4.41 (−3.37 to 12.20) n.s.	1.11	1.29

*α = 10%, *z* ≥ 1.29.

In the winter of 1919, morbidity differences by apartment size and male and female gender were insignificant (Table 4). However, results disregarding gender strongly suggest that 2‐room residents had the lowest morbidity (significant at 10%). Moreover, for both men and women, those residing in 2‐room apartments had the lowest morbidity in the winter of 1919 (7%‐8%), while they had the highest morbidity in the summer of 1918 (30%‐32%).

**Table 4:** Morbidity, as percentage suffering from an influenza‐like illness, by size of apartment and gender during the third wave, January‐March 1919

Size of apartment	Risk population	ILI cases	Probability of ILI (ILI cases in % of risk population)	Probability differences (95% CI)	*z*	Probability ratios
Both genders						
1 room	2 156	241	11.18	Reference		(ref.) 1.00
2 rooms	1 936	137	7.08	−4.10 (−10.24 to 2.04)*	−1.31	0.63
3 rooms	1 123	134	11.94	0.76 (−6.45 to 7.97) n.s.	0.21	1.07
4 rooms +	1 541	159	10.32	−0.86 (−7.39 to 5.68) n.s.	−0.26	0.92
Men						
1 room	956	109	11.41	Reference		(ref.) 1.00
2 rooms	940	58	6.17	−5.23 (−14.24 to 3.77) n.s.	−1.14	0.54
3 rooms	475	50	10.52	−0.89 (−11.89 to 10.11) n.s.	−0.16	0.92
4 rooms +	599	64	10.69	−0.72 (−10.93 to 9.50) n.s.	−0.14	0.94
Women						
1 room	1 201	132	10.99	Reference		(ref.) 1.00
2 rooms	996	79	7.93	−3.06 (−11.46 to 5.34) n.s.	−0.71	0.72
3 rooms	647	84	12.98	1.99 (−7.57 to 11.54) n.s.	0.41	1.18
4 rooms +	942	95	10.09	−0.91 (−9.44 to 7.63) n.s.	−0.21	0.92

*α = 10%, *z* ≥ 1.29.

In households with 3 rooms or less during the summer, more men than women reported ILI, but these differences were insignificant (results not shown). However, when lumping respectively the 2 smallest and the 2 largest categories, there is a tendency for lower morbidity for women living in 1‐ to 2‐room apartments (3.4 percentage lower ILI, significant at 10%, *z* = −1.36), but such a gender tendency was not found for those in 3‐ to 4‐room apartments. There was also a gender morbidity crossover going from the summer to the fall. In 1‐ to 2‐room and 4‐room apartments during the fall, more women reported an ILI although the gender differences were insignificant. However, women living in 1‐ to 2‐room apartments had 2.0 percentage point higher morbidity than men had. Finally, there were no gender differences in morbidity in the winter of 1919 or for all waves combined.

The ILI probabilities were highest during the first wave (25.8%), followed by successively lower probabilities during the second (17.4%) and third waves (9.9%), being significantly lower at the 0.1% level to the second (*z* = −5.63) and the third wave (*z* = −10.17). Morbidity in the winter of 1919 was also significantly lower at the 0.1% level compared to morbidity in the fall of 1918 (*z* = −4.55). Each apartment category and gender followed the same trend of decreasing morbidity over time (results not shown): These ILI differences were significant at the 5% level or lower with the exceptions of insignificant summer‐fall differences for males and females in, respectively, 3‐ and 4‐room apartments, fall‐winter differences for males and females in 1‐ and 3‐room apartments, and summer‐winter differences for females in 3‐room apartments.

Morbidity in the summer and fall was negatively and significantly correlated only for men, morbidity in the summer and winter waves was negatively and significantly correlated only for women, and morbidity in the fall and winter waves for both men and women was negatively and significantly correlated (Table 5).

**Table 5:** Correlations in influenza‐like illness by apartment size and gender across the 3 waves, 1918‐1919

Waves	Both genders		Men		Women	
	*r*	t‐stat	*r*	t‐stat	*r*	t‐stat
First vs second	−.44***	−2.69	−.91***	−5.51	.14	0.86
First vs third	−.24	−1.43	.25	1.50	−.58***	−3.54
Second vs third	−.77***	−4.66	−.63***	−3.86	−.84***	−5.09

***P < .01.

## 4 DISCUSSION

Combining all waves and both genders, morbidity was significantly lower for those who lived in 3‐ to 4‐room apartments than for those who lived in 1‐ to 2‐room apartments, but the difference of 3.9 percentage points in ILI was not dramatic, as was also noted in the contemporary analysis.^13^ However, that study 13 did not analyze differences in morbidity by apartment size, gender, and wave; it failed to document the crossover in morbidity by apartment size and by gender going from the first to the second wave. More males and people living in small apartments fell ill during the first wave, while more females and people living in larger apartments fell ill during the second wave.

A study of the 1918 US fall wave found an *independent* negative association between SES and morbidity and a positive association between persons per room and morbidity.^12^ This negative relation between SES and morbidity was evident in all cities analyzed and sits in opposition to the positive relation between apartment size and morbidity in Bergen in the fall of 1918. This suggests that low‐SES groups were not more protected in the fall due to a greater summer wave exposure or that not all US cities experienced a summer wave.

Based on US findings,^12^ it is likely that apartment size in Bergen is a proxy for crowding and other SES factors. Crowding is related to poverty, but also directly facilitates the spread of infectious diseases.^22^ A first candidate of other SES factors is occupational differences in exposure. Residents of small apartments are likely to have working‐class occupations with higher exposure than do residents in larger apartments with middle‐/upper‐class occupations. Prior research for Norway showed that those who fell ill during the summer were transport, hotel, and industry workers.^23^

A second candidate is socioeconomic variation in families exposed to the summer wave. In several Norwegian cities, those who were out on holiday were not exposed, while those who remained in the city were exposed.^23^ A study of the 1918‐19 pandemic in Oslo showed that apartment size was perfectly correlated with monthly rent and household income.^8^ Income and rooms in a dwelling are often‐used indicators of SES in health studies.^22^ It is likely that more families residing in the large rather than the small apartments in Bergen could afford a summer holiday. The likelihood that the higher income families were exposed to the summer wave and gained immunity to fight the fall outbreak may therefore have been lower than for the poorer families. This hypothesis is consistent with the Oslo finding, where children from rich families on the Westside were more absent from school due to influenza in the fall than were children from poor families on the Eastside.^8^

A third candidate is SES variation in hand hygiene. A 1918 Boston survey found that a larger share of “cleaner” families had no or only 1 ILI case than did “dirtier” households.^15^ A review of influenza epidemics found that hand hygiene in community settings has the potential to reduce influenza transmission.^24^ The health authorities in Bergen urged people to wash their hands and homes. This precautionary information was printed in newspapers and on posters in 1918, but probably fewer of the poor were aware of the importance of the messages.^23^ In Oslo, there was a strong negative correlation between 1918 influenza mortality and the availability of (richer) households with a bathroom.^8^ Having a bathroom is therefore likely to be positively associated with hand hygiene and negatively with morbidity. It is more likely that those residing in larger Bergen apartments had bathrooms compared to those in smaller apartments. This conjecture could therefore also possibly explain why the higher SES groups in Bergen had lower ILI in the summer of 1918.

Crowding, occupational exposure, summer holiday separation in higher SES groups, and hand hygiene are plausible mechanisms for differential SES exposure and transmission. However, individuals of lower SES may also have poorer immune function, increasing their risk of developing influenza given exposure. For example, lower SES groups were more likely to become ill when experimentally exposed to both rhino‐ and influenza viruses.^25^

The current analysis controlled for apartment size, gender, and wave, but not age and boroughs. Because individual‐level data for Bergen are not available, a multivariate analysis controlling for family size, which households and household members who had or had no ILI, and other confounders was not an option. For example, we cannot be certain whether it was high‐SES individuals who lived alone who account for the majority of ILI cases in 1‐room apartments, rather than large and low‐SES families. However, because analyses of ILI in US cities 12 and mortality in Oslo 8 found independent effects of SES and number of rooms/persons per room, it is likely that apartment size also has an independent effect on morbidity in Bergen.

More men fell ill during the first wave, especially those in the smallest apartments, while more women fell ill in the second wave. These gender differences were only significant for those aged 20‐29 years.^21^ The gender crossovers in morbidity were also observed elsewhere in Norway 23 and Copenhagen.^26^ The first affected in Boston were the male breadwinners (47%), followed by housewives (37%), schoolchildren (11%), and children not at school (3%).^15^ An explanation for the gender difference could be that young adult male breadwinners were more likely to be exposed to influenza during the first wave at work, whether that happened in the summer or the fall, than were the mostly young adult home‐based women, leading to enhanced male protection against the following waves.^21^, ^26^ That morbidity in the summer and fall was negatively and significantly correlated only for men supports this explanation (Table 5).

The morbidity in Bergen was highest during the first wave, followed by the second and third waves. With some exceptions, all apartment size groups by gender followed the same pattern. These and other findings support the hypothesis that infection once protected against further attacks^21^: First, those living in 2‐room apartments had the highest morbidity during the summer of 1918, but the lowest morbidity in the winter of 1919. Second, correlations between first/second and later waves by apartment size by gender were negative and statistically significant in 4 of 6 cases.

Bergen shared the summer wave with other Scandinavian cities.^8^, ^27^ Exposure to early waves is one suggested reason why Scandinavian cities and East Coast cities in the United States had lower morbidity and mortality during the fall of 1918 than other places.^8^, ^21^, ^27^, ^28^ Prior immunity and thus cross‐protection between waves also explain the wave‐like behavior of the pandemic.^29^, ^30^

Survey data are best in capturing the magnitude of morbidity during the 1918‐19 pandemic. First, surveys representative of general populations are a more reliable source for calculating morbidity than survey data collected actively for subgroups,^29^ or data collected passively; case data for confined settings are not representative of general populations,^30^, ^31^ and routine notification data for general populations include only cases reported to a doctor.^27^ Second, fewer people see a doctor in mild compared with severe disease outbreaks. Consequently, surveys capture better the magnitude of the mild summer wave than routine notification data do.^13^

The Bergen survey has advantages over other surveys. First, unlike the surveys in warring nations,^12^, ^14^, ^15^ where the data for young adult men are biased because a large proportion were at war, the Bergen survey derives from a neutral country not biased by the war. Second, data from Bergen are available for 3 waves, while the US and British surveys collected data only for the fall wave or published data for all waves in combination.

Two weaknesses of this study are that data are not available at an individual level and it is impossible to tease out what apartment size is a proxy for in the associations with ILI. Survey data have further limitations. The ILI cases were self‐reported and not laboratory‐confirmed. Some cases could therefore have been confused with respiratory diseases other than influenza.

Several theoretical implications arise from this study. First, studying all waves in combination masks fundamental differences in morbidity. Morbidity in Bergen was higher for lower SES groups for all waves combined. However, results showed a SES crossover in morbidity going from the summer to the fall, never before documented in the literature. In the summer, the poor had the highest morbidity, while in the fall, the rich had the highest morbidity.

Second, the results from current and other analyses of survey data with similar results 12 suggest that the socioeconomic variation in mortality found in studies using population notification data 1, 6, 7, 8, 9, 10, 11 is a product of socioeconomic differences in fatality, but also socioeconomic differences in exposure to the virus and/or development of disease given exposure.

## 5 CONCLUSIONS

The poor came down with influenza first and were overall most affected, while the rich with less exposure in the first wave tended to have higher morbidity in the second wave. This finding is concurrent with prior studies documenting that the poor had the highest 1918 pandemic mortality.^1^, ^6^, ^7^, ^8^, ^9^, ^10^, ^11^ Although this study could not tease out the mechanisms for the SES crossover in morbidity, results suggest that preparedness plans should consider how (non‐)pharmaceutical interventions can hinder socioeconomic morbidity disparities in future pandemics. Surprisingly however, social inequalities in pandemic outcomes do not form part of the discussion in international preparedness plans for pandemic influenza.^32^ This is not conducive to achieving the international goals of eradicating poverty, reducing social inequalities and ensuring good health for all by 2030.

## CONFLICT OF INTEREST

None.
